# Spinal Sagittal Alignment Assessment and Hip Range of Motion in Ambulatory Boys with Duchenne Muscular Dystrophy: Reliability, Diagnosis and Implications for Physiotherapy Management

**DOI:** 10.3390/healthcare13192392

**Published:** 2025-09-23

**Authors:** Agnieszka Stępień, Katarzyna Maślanko, Weronika Kruk-Majtyka, Grzegorz Gargas

**Affiliations:** 1 Faculty of Rehabilitation, Józef Piłsudski University of Physical Education, 00-968 Warsaw, Poland; 2 Orthos Functional Rehabilitation Center, 02-793 Warsaw, Poland; 3 Fizjoklinika Warszawskie Centrum Rehabilitacji i Osteopatii Medycznej, 02-002 Warsaw, Poland; 4 Faculty of Health Sciences, Andrzej Frycz Modrzewski Krakow University, 30-705 Krakow, Poland; 5 Department of Neurological Rehabilitation with a Subdivision of Systemic Rehabilitation, Ludwik Rydygier Memorial Specialized Hospital, 32-820 Krakow, Poland

**Keywords:** Duchenne muscular dystrophy, spinal curvature, hip joints

## Abstract

Background/Objectives: Duchenne muscular dystrophy (DMD) leads to postural abnormalities and increased lumbar lordosis, which may affect gait and spinal load. This study aimed to assess the reliability of sagittal spinal curvature measurements using the Rippstein plurimeter and to analyze spinal curvature in ambulant boys with DMD compared to healthy peers. Additionally, the study examined the effect of lower limb positioning in standing on sagittal spinal alignment in boys with DMD and investigated the relationship between hip adduction and extension range and spinal alignment. Methods: The study included 42 boys with DMD and 36 healthy peers aged 5–14 years. In boys with DMD, spinal curvature was measured using the Rippstein plurimeter in two positions: feet in alignment with hip joints axis and with feet together. In healthy participants, measurements were taken in the first position only. Hip adduction and extension ranges were also assessed in both groups. Results: Plurimeter measurements demonstrated high reliability. Boys with DMD showed significantly increased cervical retraction, greater sternal deviation from the vertical, and increased lumbar lordosis compared to healthy peers. Lower limb positioning (adduction) altered sagittal spinal alignment. Hip adduction and extension ranges were decreased in the DMD group and showed a correlation with spinal alignment. Conclusions: The Rippstein plurimeter provides reliable measurements and is useful for monitoring posture in boys with DMD. Reduced hip mobility and lower limb positioning influence lumbar lordosis and should be considered in physiotherapy planning for DMD.

## 1. Introduction

Duchenne muscular dystrophy (DMD) is a rare progressive neuromuscular disease with a genetic basis that leads to a gradual deterioration of physical performance and quality of life. The disease is caused by mutations in the gene encoding dystrophin, a protein that provides the strength, stability, and functionality of muscle fibers in healthy individuals [1,2,3]. In the natural course of the disease, the following stages are distinguished: pre-diagnosis/at diagnosis, early ambulatory phase, late ambulatory phase, early non-ambulatory phase, and late non-ambulatory phase. [1] DMD is characterized by various typical symptoms, including difficulties with walking, climbing stairs, changing positions, and postural abnormalities [1,2,3,4]. Among these, increased lumbar lordosis and anterior pelvic tilt are common [4,5], potentially affecting standing posture, gait, and contributing to lumbosacral pain [5,6].

DMD management includes the use of corticosteroids, typically initiated after age 3 [7], and physiotherapy. Physiotherapy planning should be based on an accurate clinical examination that includes reliable methods of evaluation. An essential part of the examination is postural assessment [1], including spinal alignment.

Radiographic examination is considered the gold standard for assessing spinal structures, including sagittal curvatures [8]. However, radiographic imaging is not always feasible or recommended for frequent use [9]. Alternative methods, such as using the Rippstein plurimeter to measure sagittal spinal deviations, can be treated as an additional test, particularly valuable in the everyday clinical practice of physiotherapists. Plurimeter-based assessments have been applied in healthy adolescents [10], adolescents with scoliosis [11], and for evaluating cervical spine motion and hip joint range of motion [12,13,14,15,16].

The objectives of this study were:

1. To assess inter- and intra-rater reliability of sagittal spinal curvature measurements in ambulant boys with DMD.
2. To compare sagittal spinal curvature between boys with DMD and healthy peers.
3. To analyze the effect of lower limb positioning (parallel vs. feet together) on sagittal spinal alignment in boys with DMD.
4. To examine the relationship between hip adduction/extension range and sagittal spinal alignment in boys with DMD, and compare hip joint mobility between the DMD and control groups.

## 2. Materials and Methods

### 2.1. Study Design and Ethical Approval

This research was conducted as part of a project approved by the Senate Research Ethics Committee of the University of Physical Education (SKE, 01-10/2017). Data collection took place during workshops organized by the Salemander Foundation, which supports individuals with DMD, and at a center specializing in the physiotherapy of children with neuromuscular disorders. Information about the study was disseminated via the Foundation’s social media channels. Additionally, boys with DMD receiving care at the physiotherapy center and healthy boys attending routine postural assessments were informed about the opportunity to participate.

### 2.2. Participants

The study included ambulant boys with DMD (DMD group–DMD) and age-matched healthy controls (Control group–CG). Inclusion criteria for the DMD group were: age 5–14 years, genetically confirmed diagnosis of DMD, independent ambulation, ability to maintain a standing position for at least one minute (measurement duration), no pain, no history of fractures, and the ability to understand and follow instructions. Boys who did not meet the age criterion, had difficulty maintaining a stable standing position for at least 1 min, with a history of fractures, injuries, and past musculoskeletal surgeries, were not eligible for the study.

The control group consisted of boys aged 5–14 years with no history of neurological, genetic, or metabolic disorders, no fractures or injuries in the past year, the ability to follow instructions, and a trunk rotation angle of 0–3° during the forward-bending test in the standing position. Assessment and qualification of healthy boys were conducted as part of routine periodic physiotherapy follow-up consultations focused on postural evaluation, and not during therapeutic sessions.

### 2.3. Measurement Procedures

Reliability of spinal curvature measurements was evaluated by three physiotherapists. One had 25 years of clinical experience, another 10 years, and the third had one year of professional experience. Each physiotherapist performed and recorded measurements independently. Due to the participants’ difficulty maintaining prolonged standing, each boy was assessed once by each rater at several-minute intervals. The most experienced examiner repeated the measurements at the beginning and end of the sequence.

**Figure 1:**
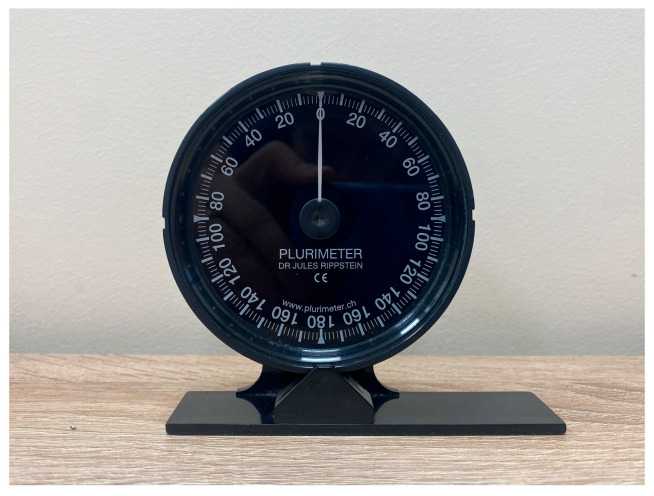
Rippstein plurimeter.

**Figure 2:**
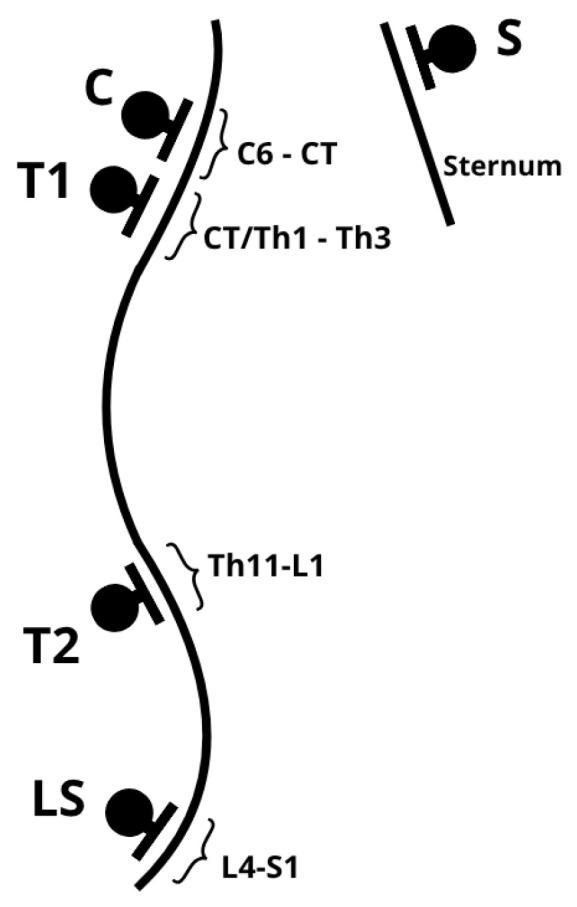
Rippstein plurimeter application points. C: Cervical spine with cervicothoracic junction (C6–CT), T1: Cervicothoracic junction with the upper thoracic spine (CT/Th1–Th3), T2: Lower thoracic spine with thoracolumbar junction (Th11–L1), LS: Lumbosacral junction (L4–S1), S: Upper sternum.

**Figure 3:**
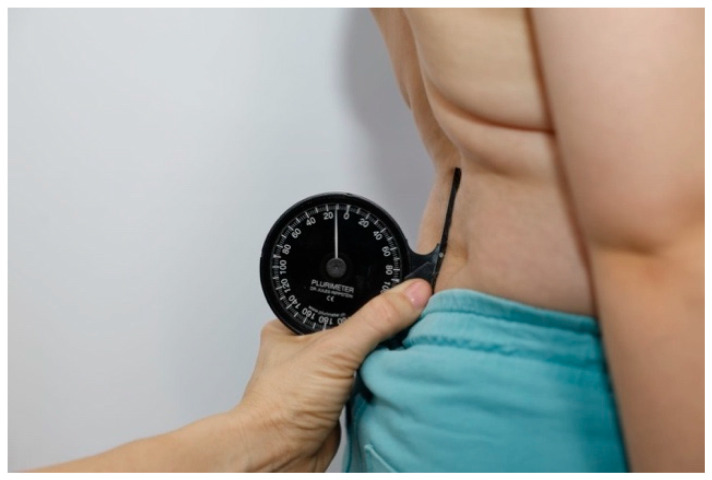
Lumbosacral junction (LS) measurement.

Measurements were performed in a standing position with lower limbs parallel, feet aligned with the hip and knee joints. The Rippstein plurimeter (Figure 1), calibrated to vertical, was used for spinal curvature assessment. The base of the plurimeter was placed at the following levels: C: Cervical spine with cervicothoracic junction (C6–CT), T1: Cervicothoracic junction with the upper thoracic spine (CT/Th1–Th3), T2: Lower thoracic spine with thoracolumbar junction (Th11–L1), LS: Lumbosacral junction (L4–S1), S: Upper sternum (Figure 2, Figure 3).

**Figure 4:**
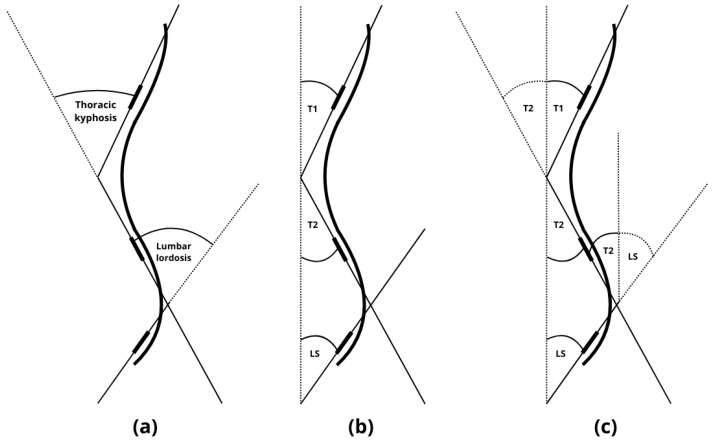
(**a**) Measurement when zeroing the plurimeter at each spinal transition level and subsequently recording the measurement at the top of both the lordotic and kyphotic curvatures. (**b**) Measurement of the angular deviations at T1, T2, and LS from the vertical axis (**c**). The sum of the two angular deviations from the vertical (T1 + T2 and T2 + LS) axis is mathematically equivalent to the values obtained by zeroing the plurimeter at each spinal transition level. T1: measurement of the angular deviations at cervicothoracic junction with the upper thoracic spine (CT/Th1–Th3), T2: measurement of the angular deviations at lower thoracic spine with thoracolumbar junction (Th11–L1), LS: measurement of the angular deviations at lumbosacral junction (L4–S1).

Placement for T1, T2, and LS was performed according to established protocols from prior studies [10,11]. Measurements were taken with the examiner standing to the right of each participant, using the left arm. To minimize examination time and compensate for the difficulty maintaining posture, the plurimeter was zeroed only once vertically, and not at each spinal transition level (LS, thoracolumbar, cervicothoracic), as recommended by Kluszczyński et al. [10]. Thoracic kyphosis was calculated by summing the angular deviations at T1 and T2, while lumbar lordosis was calculated using T2 and LS values. The sum of the two angular deviations from the vertical axis is mathematically equivalent to the values obtained by zeroing the plurimeter at each spinal transition level and subsequently recording the measurement at the top of both the lordotic and kyphotic curvatures. This relationship is illustrated in Figure 4.

The next stage involved measuring spinal curvature in the DMD group in two standing positions: feet aligned with the hip joint axis (parallel stance) (Position 1) and feet together (hip adduction) (Position 2). In CG, measurements were taken in Position 1. Spinal alignment was compared between the DMD and CG, and between Position 1 and Position 2 within the DMD group.

Hip adduction was measured with the participant in side-lying, with the non-tested limb (closer to the ground) flexed at the hip and knee joint. The tested leg was extended at the knee and positioned neutrally in the sagittal plane. The examiner stabilized the pelvis and adducted the upper leg toward the table, using the Rippstein plurimeter placed on the lateral surface of the thigh, just above the knee joint. The device was zeroed to horizontal. Hip extension range was measured following established protocols [12,17], with the plurimeter placed on the anterior thigh just above the patella.

### 2.4. Statistical Analysis

Data analysis was conducted using IBM SPSS Statistics 20, Chicago, IL, USA. Means and standard deviations were calculated. To assess reliability, the intraclass correlation coefficient (ICC) and 95% confidence intervals were determined. ICC interpretation was as follows: <0.5 = poor reliability, 0.5–0.75 = moderate reliability, 0.75–0.9 = good reliability, 0.9 = excellent reliability [18]. Due to non-normal distribution, non-parametric tests were used: Mann–Whitney U test to compare sagittal curvature parameters between groups, Wilcoxon signed-rank test to compare spinal alignment in DMD group between Positions 1 and 2, Mann–Whitney U test to compare hip range of motion between groups and Spearman correlation: to evaluate relationships between sagittal spinal parameters and hip ROM in the DMD group. A significance level of *p* < 0.05 was used.

## 3. Results

### 3.1. Reliability of Spinal Curvature Measurements with the Rippstein Plurimeter

A total of 40 ambulant boys with DMD aged 5–14 years participated in the reliability assessment of sagittal spinal curvature measurements. The analysis demonstrated a high level of measurement reliability. The intraclass correlation coefficient (ICC) among the three raters ranged from 0.897 to 0.972, while intra-rater reliability for the most experienced examiner ranged from 0.867 to 0.953. Detailed results are presented in Table 1.

### 3.2. Comparison of Sagittal Spinal Alignment Between the DMD and Control Groups

Sagittal spinal curvature measurements were analyzed for 42 boys with DMD and 36 healthy boys. Detailed data are presented in Table 2. There were no significant differences in age or body mass between the groups. Boys with DMD were shorter and had a higher BMI compared to their healthy peers. Comparison of spinal alignment in both groups, with feet positioned in line with the hip joints, revealed significant differences in the alignment of the cervical spine (greater cervical retraction in DMD), lumbosacral junction (greater anterior tilt of the sacrum in DMD), sternum (greater deviation from the vertical in DMD), and lumbar lordosis (greater in DMD) (Table 2). 

In Position 1, the range of angular deviations (min–max) of the sternum, C, T1, T2, and LS among individual DMD participants ranged from 28° to 47°, while in the control group the range was 16° to 20°. Thoracic kyphosis varied from –6.00° to 52.00°, and lumbar lordosis from 20.00° to 70.00° in the DMD group. In the control group, thoracic kyphosis ranged from 14° to 36°, and lumbar lordosis ranged from 18° to 44° (Table 2).

### 3.3. Effect of Lower Limb Position on Sagittal Spinal Alignment in Boys with DMD (Position 1: Parallel Legs vs. Position 2: Feet Together with Hip Adduction)

Comparison of spinal alignment in the two positions revealed significant differences in the T1, T2, and LS segments. Cervical spine and sternal alignment did not differ significantly but showed a trend toward change. Standing with feet together (Position 2) induced an increase in lumbar lordosis and did not affect thoracic kyphosis. Specifically, hip adduction increased the average lumbosacral junction tilt by 4° and lumbar lordosis by more than 7°. A trend toward head and cervical retraction was also observed in Position 2 (Table 3).

Within the DMD group, a wide range of spinal deviation values was observed in both standing positions (Table 3). For example, the difference between the minimum and maximum LS angle was 50° in Position 1, and 52° in Position 2. Among healthy boys (Table 2), the corresponding difference in LS was 20°, indicating high variability in spinal curvature within the DMD group.

#### Comparison of Sagittal Spinal Alignment by Age in the DMD and Control Groups

Additional analysis was conducted based on age. The DMD group was divided into two subgroups: 4–7 years (*n* = 19) and 7.1–14 years (*n* = 23). The control group was similarly divided: 4–7 years (*n* = 17) and 7.1–14 years (*n* = 19). In the DMD group (Position 1), no significant differences were found between age subgroups in the alignment of the C segment (*p* = 0.808), T1 (*p* = 0.439), sternum (*p* = 0.155), or thoracic kyphosis (*p* = 0.249).

However, older boys had significantly greater angular values in T2 (*p* = 0.019), LS (*p* = 0.042), and lumbar lordosis (*p* = 0.002). In Position 2, older boys with DMD had significantly lower T1 angles (*p* = 0.047), lower LS tilt (*p* = 0.002), and greater lumbar lordosis (*p* = 0.002). Cervical alignment (*p* = 0.665), T2 (*p* = 0.136), sternal tilt (*p* = 0.258), and thoracic kyphosis (*p* = 0.361) did not differ significantly between age groups. Thus, regardless of lower limb position, older boys exhibited greater LS tilt and increased lumbar lordosis compared to younger boys.

In the control group, comparison of younger (*n* = 17) and older (*n* = 19) participants revealed no significant differences, except for a change in sternal alignment (*p* = 0.044).

### 3.4. Hip Joint Range of Motion and Correlation with Sagittal Spinal Alignment

Significant differences were found in hip extension range (both left and right) between the DMD and control groups (Table 2), with boys with DMD exhibiting reduced hip extension. They also demonstrated significantly reduced adduction in both hips compared to healthy peers. Age-based analysis within the DMD group showed that older boys had significantly reduced extension in the left hip (*p* = 0.024), and reduced adduction in both the left (*p* < 0.001) and right (*p* < 0.001) hips. 

Both age and body height were significantly correlated with hip extension and adduction (excluding HER). Spinal segments T1, T2, and LS showed strong correlations with lumbar lordosis, with T1 displaying a negative correlation (Table 4).

## 4. Discussion

One of the factors that negatively affects gait quality and other daily activities in boys with DMD is increased lumbar lordosis [5]. Previous literature suggests that lumbar lordosis increases with age in boys with DMD [5,19]. In the present study, sagittal spinal alignment in ambulant boys with DMD was assessed and compared to that of healthy peers. The Rippstein plurimeter, previously used to assess sagittal spinal alignment in young adults [20,21], individuals over 55 years of age [22], children and adolescents [10,23,24], adolescents with scoliosis [11], and to evaluate cervical spine and hip joint range of motion [12,13,14,15,16], as well as neurodynamic tests [11], was used.

### 4.1. Measurement Reliability

Filiz et al. evaluated the inter-rater reliability of sagittal curvature measurements in five individuals with DMD and five healthy controls using a digital inclinometer. Measurements were taken twice, one week apart, by an examiner with 10 years of experience using the instrument. The results demonstrated excellent repeatability [5]. Previously, the reliability of spinal curvature measurements with the Rippstein plurimeter in boys with DMD has not been evaluated. This gap justified one of the primary goals of this study. The present study, conducted by three raters on a sample of 40 participants, revealed high ICC values, indicating that the Rippstein plurimeter can be reliably used in clinical practice. Spinal curvature measurements should be incorporated into routine diagnostics in boys with DMD to support physiotherapy planning and evaluate intervention outcomes.

### 4.2. Increased Lumbar Lordosis

Baptista et al. found significant differences in pelvic positioning between DMD and non-DMD groups, with greater anterior pelvic tilt and anterior displacement of the center of mass in the DMD group [25]. The values of lumbar lordosis were found to be significantly higher by an average of 6.9 degrees in the DMD group compared to healthy subjects. The values of thoracic kyphosis did not differ significantly between the groups [5]. The current findings support these results, showing that boys with DMD present significantly increased lumbar lordosis in the standing position with feet aligned to the hip axis, compared to healthy peers. The observed difference in lumbar lordosis (mean increase of 6.88°) closely matches the results reported by Filiz et al. [5]. However, the current study was conducted on a larger sample (36 healthy, 42 DMD participants) than previous studies (Filiz [5]: 10 healthy, 29 DMD; Baptista: 10 healthy, 10 DMD), providing more robust evidence.

Spinal alignment changed with age. In boys over age 7, there were significantly higher angular values in T2, LS, and lumbar lordosis. These results suggest a need for early preventive interventions and assessment of their effectiveness, ideally beginning in preschool years. Filiz et al. reported a moderate positive correlation between lumbar lordosis and age [5]. Increased lumbar lordosis may also relate to other functional aspects. In the study by Filiz, lumbar lordosis was strongly and negatively correlated with the 6-minute walk test (6MWT), moderately and positively correlated with the Timed Up and Go (TUG) and Time to Rise from Floor (TRF) tests, and moderately and negatively correlated with the Berg Balance Scale (BBS). Boys with lordosis greater than 36° performed significantly worse on the BBS, TUG, and muscle strength assessments (MMT) for both upper and lower limbs, and they generally showed lower functional performance [5].

Vandekerckhove et al. identified three gait patterns in individuals with DMD. Regardless of gait pattern, all showed increased anterior pelvic tilt during the stance phase [26]. Romano et al. observed that boys with DMD had greater anterior pelvic tilt, greater hip abduction, and wider step width during gait compared to healthy peers [27]. While a wider step width may result from unstable gait, it may also be caused by restricted hip adduction, as identified in the present study. Further research is warranted in this area.

Large variability in sagittal spinal angles (min–max) in the DMD group compared to controls was found in this study. Differences in spinal curvature and total values of thoracic kyphosis and lumbar lordosis reached dozens of degrees in the DMD group. This wide range underscores the importance of regular spinal curvature assessment in boys with DMD to guide individualized physiotherapy programs.

### 4.3. Hip Adduction Increases Anterior Pelvic Tilt and Lordosis

This study found that standing with feet together, which induces hip adduction, increased the anterior tilt of the lumbosacral junction by over 4° and lumbar lordosis by more than 7°. The relationship between hip adduction and increased anterior pelvic tilt and lumbar lordosis in the sagittal plane may influence gait quality. During the loading response and mid-stance phases of gait, the hip moves toward adduction as the center of mass shifts over the support limb. According to researchers, increased lumbar lordosis serves as a compensatory strategy in response to gluteal muscle weakness. In DMD, pelvic instability results from this weakness, and compensatory tension in the iliotibial band often causes anterior pelvic tilt, positioning the trunk in a lordotic posture. This altered posture can displace the center of mass and contribute to balance and gait difficulties [6,28]. Increased anterior pelvic tilt and lumbar lordosis make it harder to maintain single-limb support during the mid-stance phase of gait. This study aimed to clarify the origin of this relationship. The findings suggest that increased lumbar lordosis may result from limited hip adduction. Changes in lower limb frontal plane alignment (abduction–adduction) during standing affect sagittal plane spinal alignment. Future studies should investigate whether reducing hip abductor contractures can prevent increased lumbar lordosis during standing with feet together. They could include plurimeter measurements in the sitting position and assess trunk muscle strength to better differentiate the contributions of hip contractures and truncal weakness to sagittal spinal alignment. This research highlights the importance of detailed functional assessment in boys with DMD.

### 4.4. Hip Range of Motion Decreases with Age

The study revealed significantly reduced hip extension and adduction in older boys. This finding supports the implementation of stretching programs before age 7 to prevent ROM restrictions. Willcocks et al. demonstrated that individuals with DMD exhibited restricted hip extension compared to healthy norms. Although these contractures progressed slowly during the ambulant stage, progression accelerated upon transitioning to the non-ambulant stage, with only a weak correlation with age [29]. Choi et al. found that hip flexion contractures were rare in ambulant individuals with DMD but became more common and severe in non-ambulant individuals [30]. In boys with DMD, analysis of data on hip extension and hip adduction range of motion suggested a subtle downward trend with age. Hip extension deficits appeared early, even in young children. Extension ROM declined at a rate similar to that in healthy children. However, adduction ROM declined more rapidly, despite no major early deficits, indicating that abduction contractures may not appear early but develop after loss of ambulation [31].

Many researchers have analyzed gait kinematics, showing that children with DMD have reduced peak hip extension angles, regardless of walking speed [32]. Pelvic tilt during gait increases over time in individuals with DMD, possibly due to increased stiffness and shortening of the hip flexors and/or weakened hip extensors [6,32,33]. As a result, hip extension deficits during gait increase by nearly 2° per year [34].

Reduced flexibility in the hamstrings, gastrocnemius, hip flexors, and tensor fasciae latae has been shown to correlate significantly with functional performance, as measured by the 6-minute walk test [35]. Another study reported that boys with greater lower limb ROM limitations had poorer outcomes in the 6MWT and 10-meter walk/run. Better walking function was associated with greater ROM in the hip, knee, and ankle joints—though this did not imply a causal relationship. It remains unclear whether lower limb contractures directly cause decreased function or whether both progress in parallel with disease severity [29].

### 4.5. Correlations

The T1 segment, not T2, showed the strongest correlation with lumbar lordosis. This region contains several muscles involved in scapular stabilization and upper limb function. Some studies have examined the relationship between thoracic kyphosis and upper extremity function and motor performance in non-ambulant patients with DMD [36]. Future research should examine these relationships in ambulant boys as well.

### 4.6. Study Limitations

This study did not examine the relationship between spinal curvature and functional status, which could provide valuable context. Future longitudinal studies with larger cohorts, dynamic and multi-planar assessments, and the inclusion of muscular strength, functional performance, and daily physical activity measures would enhance the clinical relevance of these findings. These factors may affect postural control and could introduce variability in measurements. Spinal curvature was measured using the Rippstein plurimeter, although radiography remains the gold standard for spinal assessment. Plurimeter measurements were not validated against radiographic assessments in the study group; however, previous research has validated another type of mechanical inclinometer in different populations [37,38]. These findings emphasize the need for further research to directly compare plurimeter measurements with radiographic standards in this population. A limitation of our study is the absence of spinal curvature assessment with feet together in the control group. Since measurements in boys with DMD showed an increase in lumbar lordosis in this position, it remains unclear whether this reflects a disease-specific phenomenon or a general characteristic of pediatric sagittal alignment. This uncertainty emphasizes the importance of cautious interpretation and indicates that additional comparative data would be particularly valuable. Further studies, including healthy controls, are required. Finally, the cross-sectional design precludes conclusions regarding the progression of postural changes over time, highlighting the need for longitudinal studies. Furthermore, although all controls were free from musculoskeletal or neurological disorders, data on habitual physical activity were not systematically collected. Collecting such information in future research could provide additional insights and enhance the clinical relevance of the findings.

## 5. Conclusions

Sagittal spinal alignment measurements using the Rippstein plurimeter are reliable and can be effectively used in clinical practice. Changes in lower limb positioning in the frontal plane (abduction–adduction) while standing significantly influence sagittal spinal alignment. In boys with DMD, there is a clear relationship between hip adduction and sagittal alignment of the spine and pelvis. Specifically, hip adduction may contribute to increased anterior pelvic tilt and lumbar lordosis. This relationship requires further investigation. 

## Figures and Tables

**Table 1:** Interobserver and intraobserver reliability (ICC) for measurements of the spine in the sagittal plane and associated 95% confidence intervals in the DMD group (*n* = 40).

Parameter	Interobserver Reliability			Intraobserver Reliability		
	ICC	95% Confidence Interval	*p*	ICC	95% Confidence Interval	*p*
**Sternum**	0.947	0.910–0.970	<0.001	0.939	0.888–0.967	<0.001
**C**	0.897	0.825–0.942	<0.001	0.878	0.781–0.934	<0.001
**T1**	0.972	0.951–0.984	<0.001	0.947	0.904–0.972	<0.001
**T2**	0.915	0.857–0.952	<0.001	0.867	0.761–0.928	<0.001
**LS**	0.957	0.928–0.976	<0.001	0.953	0.914–0.975	<0.001

C: Cervical spine with cervicothoracic junction, T1: Cervicothoracic junction with the upper thoracic spine, T2: Lower thoracic spine with thoracolumbar junction, LS: Lumbosacral junction, S: Upper sternum

**Table 2:** Comparison of spinal alignment in the sagittal plane in a group of boys with Duchenne dystrophy (DMD) and healthy peers (CG). Position 1 Standing-feet in line with hip joints (*n* = 42).

		DMD (*n* = 42)			CG (*n* = 36)		DMD vs. CG
Parameter	Mean ± SD	Median	Range	Mean	Median	Range	*p*
Age (years)	7.98 ± 2.57	8.00	5.00–14.00	8.10 ± 2.32	8.00	5.00–12.5	0.651
Body weight (kg)	27.99 ± 8.84	26.00	16.50–51.00	31.15 ± 10.68	28.00	19.00–55.00	0.176
Body height (cm)	124.00 ± 11.88	124.00	102.00–152.00	134.87 ± 15.31	132.5	112.00–168.00	**0.002**
BMI (kg/m^2^)	17.73 ± 2.69	17.20	12.93–24.33	16.71 ± 2.49	15.91	13.88–21.37	**0.037**
Steroid treatment	30/42 (71.4%)	-	-	-	-	-	-
Scoliosis	6/42 (14.3%)	-	-	-	-	-	-
Ability to walk up stairs	37/42 (88.1%)	-	-	-	-	-	-
Number of falls per week	35/42 (83.3%)	2	1–35	-	-	-	-
Physical therapy	40/42 (95.2%)	3	1–5	-	-	-	-
**Position 1:** **Standing-feet in line with hip joints**							
Sternum (°)	32.38 ± 8.96	32.00	12.00–52.00	27.25 ± 4.65	26.5	20.00–38.00	**0.002**
C (°)	17.02 ± 7.15	18.00	0.00–30.00	19.39 ± 3.14	20.00	10.00–26.00	**0.017**
T1 (°)	10.90 ± 8.96	10.00	−15.00–32.00	11.33 ± 3.80	10.00	4.00–20.00	0.792
T2 (°)	13.48 ± 6.62	13.00	0.00–28.00	11.55 ± 3.51	10.00	4.00–20.00	0.120
LS (°)	23.76 ± 8.71	24.00	10.00–48.00	18.61 ± 4.99	20.00	6.00–26.00	**0.004**
Thoracic kyphosis (°)	24.38 ± 8.82	24.00	−6.00–52.00	23.14 ± 5.37	22.00	14.00–36.00	0.570
Lumbar lordosis (°)	37.24 ± 11.45	36.00	20.00–70.00	30.36 ± 6.33	31.00	18.00–44.00	**0.005**
HEL	14.50 ± 8.03	17.00	−8.00–26.00	18.94 ± 4.21	20.00	4.00–26.00	**0.024**
HER	14.26 ± 6.55	12.00	0.00–26.00	17.83 ± 5.10	20.00	4.00–26.00	0.352
HADL	28.67 ± 8.53	30.00	8.00–40.00	36.33 ± 3.11	36.00	28.00–40.00	**<0.001**
HADR	29.14 ± 8.53	30.00	10.00–40.00	36.61 ± 2.92	36.50	28.00–42.00	**<0.001**

C: Cervical spine with cervicothoracic junction, T1: Cervicothoracic junction with the upper thoracic spine, T2: Lower thoracic spine with thoracolumbar junction, LS: Lumbosacral junction, S: Upper sternum, HE—hip extension; HAD—hip adduction; L—left, R—right.

**Table 3:** Comparison of spinal alignment in the sagittal plane in boys with DMD (*n* = 42) in the standing position—feet in line with hip joints (position 1) and feet together (position 2).

	Position 1 Feet in line with hip joints			Position 2 Feet together			Position 1 vs. Position 2
Parameter	Mean ± SD	Median	Range	Mean ± SD	Median	Range	*p*
Sternum (°)	32.38 ± 8.96	32.00	12.00–52.00	33.38 ± 8.04	34.00	18.00–50.00	0.067
C (°)	17.02 ± 7.15	18.00	0.00–30.00	15.79 ± 7.38	16.00	0.00–30.00	0.069
T1 (°)	10.90 ± 8.96	10.00	−15.00–32.00	7.67 ± 10.74	9.00	−15.00–40.00	**<0.001**
T2 (°)	13.48 ± 6.62	13.00	0.00–28.00	16.43 ± 7.00	16.00	2.00–30.00	**0.001**
LS (°)	23.76 ± 8.71	24.00	10.00–48.00	28.07 ± 10.19	25.50	10.00–52.00	**<0.001**
Thoracic kyphosis (°)	24.38 ± 8.82	24.00	−6.00–52.00	23.43 ± 10.12	22.00	−6.00–52.00	0.535
Lumbar lordosis (°)	37.24 ± 11.45	36.00	20.00–70.00	44.50 ± 13.26	43.00	22.00–74.00	**<0.001**

C: Cervical spine with cervicothoracic junction, T1: Cervicothoracic junction with the upper thoracic spine, T2: Lower thoracic spine with thoracolumbar junction, LS: Lumbosacral junction, S: Upper sternum.

**Table 4:** Correlations between postural parameters in the sagittal plane and the ranges of hip extension and adduction in the DMD group (*n* = 42). Position 1.

	Age	Height	Weight	S	C	T1	T2	LS	K	L
**S**	0.110	0.127	0.305 *	-	−0.629 **	−0.593 **	0.578 **	0.066	−0.238	0.422 **
***p***	0.488	0.423	0.050		0.000	0.000	0.000	0.678	0.129	0.005
**C**	0.089	0.086	0.017	−0.629 **	-	0.675 **	−0.307 *	−0.050	0.516 **	−0.246
	0.574	0.589	0.917	0.000		0.000	0.048	0.753	0.000	0.116
**T1**	−0.081	−0.099	−0.153	−0.593 **	0.675 **	-	−0.405 **	−0.488 **	0.676 **	**−** **0** .612 **
	0.611	0.532	0.333	0.000	0.000		0.008	0.001	0.000	**0.000**
**T2**	0.268	0.269	0.300	0.578 **	−0.307 *	−0.405 **	-	0.087	0.337 *	**0** .678 **
	0.086	0.085	0.054	0.000	0.048	0.008		0.582	0.029	**0.000**
**LS**	0.425 **	0.355 *	0.345 *	0.066	−0.050	−0.488 **	0.087	-	−0.404 **	**0** .772 **
	0.005	0.021	0.025	0.678	0.753	0.001	0.582		0.008	**0.000**
**K**	0.167	0.126	0.064	−0.238	0.516 **	0.676 **	0.337 *	−0.404 **	-	−0.098
	0.289	0.426	0.688	0.129	0.000	0.000	0.029	0.008		0.538
**L**	0.504 **	0.457 **	0.480 **	0.422 **	−0.246	**−** **0** .612 **	**0** .678 **	**0** .772 **	−0.098	-
	0.001	0.002	0.001	0.005	0.116	**0.000**	**0.000**	**0.000**	0.538	
**HEL**	−0.476 **	−0.395 **	−0.324 *	0.053	−0.049	0.070	−0.180	−0.294	−0.137	−0.287
	0.001	0.010	0.037	0.741	0.760	0.658	0.253	0.059	0.388	0.066
**HER**	−0.350 *	−0.186	−0.134	0.087	−0.122	0.024	−0.077	−0.309 *	−0.087	−0.224
	0.023	0.239	0.398	0.582	0.443	0.881	0.627	0.047	0.583	0.155
**HADL**	−0.564 **	−0.408 **	−0.300	−0.015	0.018	0.019	−0.175	−0.340 *	−0.076	−0.334 *
	0.000	0.007	0.053	0.927	0.911	0.906	0.267	0.027	0.634	0.031
**HADR**	−0.513 **	−0.349 *	−0.267	−0.218	0.115	0.222	−0.322 *	−0.411 **	0.019	−0.466 **
	0.001	0.023	0.088	0.166	0.469	0.158	0.037	0.007	0.907	0.002

C: Cervical spine with cervicothoracic junction, T1: Cervicothoracic junction with the upper thoracic spine, T2: Lower thoracic spine with thoracolumbar junction, LS: Lumbosacral junction, S: Upper sternum, HE—hip extension; HAD—hip adduction; L—left, R—right, K—Thoracic kyphosis; L—Lumbar lordosis. * The correlation is significant at the 0.05 level (two-tailed). ** The correlation is significant at the 0.01 level (two-tailed).

## Data Availability

The original contributions presented in this study are included in the article. Further inquiries can be directed to the corresponding author.
